# Frenemies within: An Endocarditis Case in Behçet’s Disease

**DOI:** 10.3390/jpm11080728

**Published:** 2021-07-27

**Authors:** Diana Moroșan, Adela Șerban, Cătălin Trifan, Svetlana Encica, Sorin Pop, Tudor Costinel Șerban, Simona Rednic, Laura Damian

**Affiliations:** 1 Department of Rheumatology, “Iuliu Hatieganu” University of Medicine and Pharmacy Cluj-Napoca, 8 Victor Babeș St., 400012 Cluj-Napoca, Romania; vulcu.diana@yahoo.com (D.M.); srednic.umfcluj@gmail.com (S.R.); 2 Department of Cardiology, “Iuliu Hatieganu” University of Medicine and Pharmacy Cluj-Napoca, 8 Victor Babeș St., 400012 Cluj-Napoca, Romania; adelamserban@yahoo.com; 3 Department of Cardiology, “Niculae Stancioiu” Heart Institute Cluj-Napoca, 19-21, Calea Moților St., 400001 Cluj-Napoca, Romania; tudor_serban@ymail.com; 4 Department of Cardiovascular Surgery, “Niculae Stancioiu” Heart Institute Cluj-Napoca, 19-21, Calea Moților St., 400001 Cluj-Napoca, Romania; trifancatalin@gmail.com; 5 Department of Pathology, “Niculae Stancioiu” Heart Institute Cluj-Napoca, 19-21, Calea Moților St., 400001 Cluj-Napoca, Romania; 6 1st Internal Medicine Department, Emergency Clinical County Hospital Cluj, 3-5 Clinicilor St., 400006 Cluj-Napoca, Romania; popsorin98@gmail.com; 7 Department of Rheumatology, Centre for Rare Musculoskeletal Autoimmune and Autoinflammatory Diseases, Emergency Clinical County Hospital Cluj, 2-4 Clinicilor St., 400006 Cluj-Napoca, Romania; ldamian.reumatologie@gmail.com; 8 CMI Reumatologie Dr. Damian, 6-8 P. Maior St., 400002 Cluj-Napoca, Romania

**Keywords:** thrombotic endocarditis, Behçet’s disease, *Coxiella burneti*, *Abiotrophia defectiva*, thrombocytopenia, valve replacement, immune tolerance

## Abstract

A 57-year female patient diagnosed with Behçet’s disease, on azathioprine, was noticed to have at a routine examination antinuclear and antiphospholipid antibodies. An overlapping lupus-like syndrome was diagnosed; hydroxychloroquine and aspirin were added. Three years later, the patient presented with dyspnea and sweating, with no fever. A cardiac bruit was noted; a giant vegetation was detected by echocardiography. Laboratory revealed severe thrombocytopenia, antiphospholipid antibodies and low complement. Blood cultures were positive for *Abiotrophia defectiva* serology and also revealed a chronic *Coxiella burnetii* infection. Antibiotic therapy, low-dose anticoagulation and control of the underlying disease mildly improved the platelet count, which fully recovered only after cardiac valve replacement. However, the Behçet’s disease, initially quiescent, flared after the therapy of infections. We discuss potential links between Behçet’s disease and the occurrence of antinuclear and antiphospholipid antibodies and *Coxiella* endocarditis in this setting. We also highlight the differences between the endocarditis in Behçet’s disease, antiphospholipid syndrome, *Coxiella burnetii* and *Abiotrophia defectiva* infection, respectively. Intracellular infections may modify the presentation of autoimmune diseases. Confounding clinical features of *Coxiella* persistent infection and non-bacterial thrombotic endocarditis in Behçet’s disease warrant further insight.

## 1. Introduction

*Coxiella burnetii* (*C. burnetii*) is an obligate intracellular Gram-negative pathogen, the etiologic agent of Q fever [1]. The microorganism has a remarkable ability to live and prosper intracellularly and is associated with a plethora of autoimmune phenomena, mostly in persistent disease. Among these, antiphospholipid (APL) antibodies, mainly anticardiolipin (ACL) and anti-beta2 glycoprotein-1 (Aβ2GP1) antibodies, are notably generated during the *C. Burnetii* infection [2].

*Abiotrophia defectiva* (*A. defectiva*) is a variant of fastidious nutritionally variant streptococci, part of the commensal oral, gastrointestinal and genito-urinary flora [3]. *A. defectiva* is associated with endocarditis with large vegetations (>10 mm) and higher rate of complications and valve replacement (around 50%) [4]. *A. defectiva* infections occur especially in immunocompromised patients [4].

Right heart thrombotic endocarditis is a hallmark of Behçet’s disease (BD), a form of vasculitis at the crossroads of inflammation and thrombosis which may involve arteries as well as veins. BD pathogenesis implicates autoimmune and autoinflammatory mechanisms and is strongly associated with HLA-B51, but the disease has no specific tests [5]. Antinuclear antibodies (ANA), and anti-neutrophil cytoplasm autoantibodies (ANCA), which may be found in a minority of BD, are rather used for the differential diagnosis with systemic lupus erythematosus and other collagen-vascular diseases or vasculitides [5,6]. APL may be encountered in BD, but their contribution to the pathogenesis of BD-associated thrombosis is unclear [7]. As APL occur in infections as well, with the same clinical and biological picture—livedo reticularis, thrombocytopenia, thromboses and/or stroke- the differential diagnosis may be challenging.

We present a rare case of right heart endocarditis evolving slowly without fever in a patient with BD, in whom a tricuspid valve endocarditis with *A. defectiva* and a *C. burnetii* persistent infection were diagnosed.

## 2. Case Report

A 57-year female patient with a 10-year history of HLA- B51positive Behçet’s disease with recurrent episodes of thrombophlebitis, oral and genital aphtae and arthritis, was generally well controlled on azathioprine 100 mg/day (colchicine had not been digestively tolerated). At a routine examination she was noticed to have ANA (1/320, speckled type) and ACL positivity (55 UGPL or IgG Phospholipid Units, normal <20 UGPL), with no livedo reticularis, thrombocytopenia or other APL-associated clinical features. An overlapping lupus-like syndrome was presumed; hydroxychloroquine and low-dose aspirin were added, taken for a few months then stopped by the patient.

Three years thereafter, during the COVID-19 first pandemic wave, the patient presented for dyspnea and sweating, with no fever. A systolic murmur, best heard on left lower border sternum with an increase in intensity during inspiration, was noted. Physical examination also revealed a blood pressure of 110/70 mmHg, a heart rate of 75 beats/minute, an oxygen saturation on room air of 98%, a temperature of 36.4 °C and a BMI of 29.6 kg/m^2^ (overweight). No specific peripheral signs of infective endocarditis were found. Electrocardiogram showed a normal sinus rhythm and an incomplete right bundle branch block.

**Figure 1:**
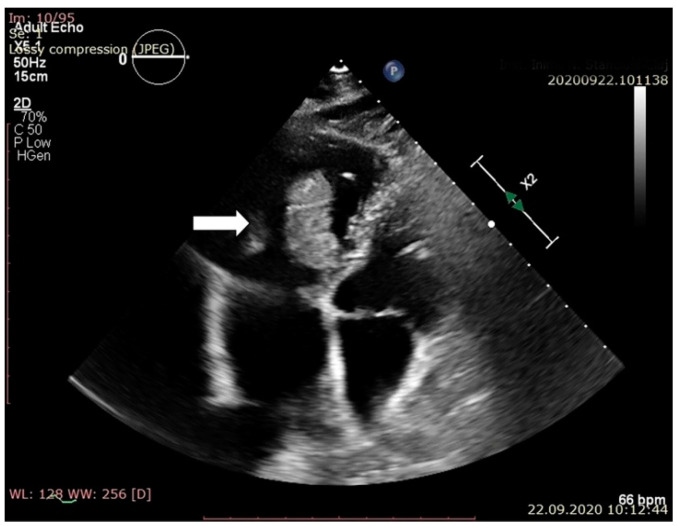
Apical 4 chambers view. Presence of the vegetation in right ventricle in diastole.

**Figure 2:**
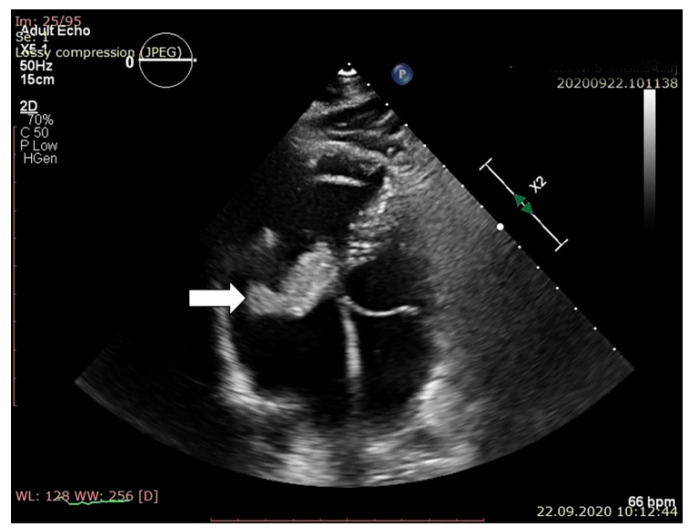
Apical 4 chamber view. Systolic prolapsed in the right atrium of the septal leaflet and vegetation.

Routine transthoracic echocardiography (TTE), performed with a Philips EPIQ 7 echo system using a 1–5 MHz probe, described two echogenic, inhomogeneous masses with a maximum diameter of 39 and 18 mm attached to the lateral and septal leaflets of the tricuspid valves. A systolic prolapse in the right atrium and a diastolic prolapse in the right ventricle of the two masses were noted (Figure 1 and Figure 2).

The color Doppler mode detected a severe tricuspid regurgitation and a significant tricuspid valve obstruction with a mean transvalvular gradient of 4 mmHg and a peak velocity of 1.4 m/s, measured by continuous wave Doppler mode. The tricuspid valve leaflets were highly thickened, rigid, with huge vegetations that practically encompassed the two cusps. Vegetations showed echogenicity similar to the myocardium with more hyperechogenic areas of fibrosis and calcification due to the subacute stage of endocarditis. Mitral and aortic valves were morphologically normal, with no significant regurgitation. General and regional systolic function of the left ventricle was normal. A contrast-enhanced cerebral, thoracic, abdominal and pelvic CT scan was performed to rule out perivalvular abscesses as well as embolizations. A partial filling defect of the lower left and right pulmonary lobar arteries was observed; cerebral and abdominal embolizations were excluded.

Laboratory tests revealed a low-grade, chronic inflammation (erythrocyte sedimentation rate) ESR 35 mm/h, C reactive protein CRP 14.92 mg/L, range ≤5 mg/L, fibrinogen 411 mg/dL, range 200–400 mg/dL), leukocytosis (white blood cell WBC 11.73 × 10^3^/μL) anemia (Hb 9.8 g/dL), thrombocytopenia (26,000/μL then 18,000 μL), a prolonged partial thromboplastin time (APTT 46.9 s, range ≤36 s), elevated D-dimers (1278 ng/mL, range <250 ng/mL), positive ACL and anti-beta2 glycoprotein (Aβ2GPI) antibodies (256 U/mL, range <20 U/mL) and lupus anticoagulant (LA), ANA 1/320 with a speckled pattern, a negative ANA extensive panel, and low C3 complement (71 mg/dL, range 90–120 mg/dL). Two blood cultures were positive for *A. defectiva* identified using the VITEK2 Compact analyzer (bioMerieux, Marcy-l’Étoile, France). Serology was also positive for *C. burnetii* anti-phase II IgG (1/128) and phase I IgG (1/1024) antibodies. Repeated RT-PCR (real-time polymerase chain reaction) for SARS-CoV2 and anti-SARS-CoV2 IgM and IgG antibodies were negative. She received ceftriaxone 4 g/day for 2 weeks, then vancomycin 30 mg/kg/day in 3 doses for 6 weeks, combined with aminoglycoside (gentamycin 3 mg/kg /day iv) for 2 weeks. Three blood cultures were repeatedly negative thereafter. Hydroxychloroquine (600 mg/day) and doxycycline 200 mg/day were continued, but the thrombocytes increased only to 22,000/μL despite the improvement of the general status, a marked reduction of the APL titer, ANA disappearance and C3 correction. Low-molecular weight heparin (nadroparin 30 IU/day), 10 mg prednisone/day, followed by azathioprine 50 to 100 mg/day were added, resulting in a further modest increase of the platelet count (30,000/μL) to allow surgery.

**Figure 3:**
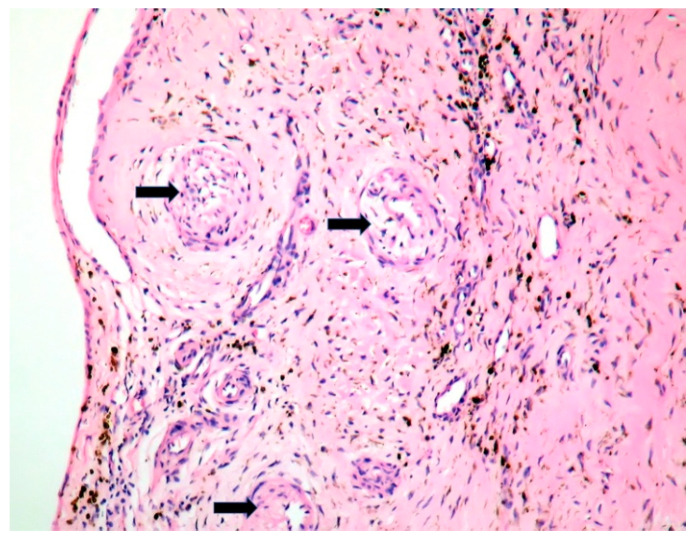
Tricuspid valve reparative changes; neoformation vessels with subocclusive intimal hyperplasia; siderophages after valve hemorrhage (Hematoxylin Eosin stain (HE) stain × 10).

**Figure 4:**
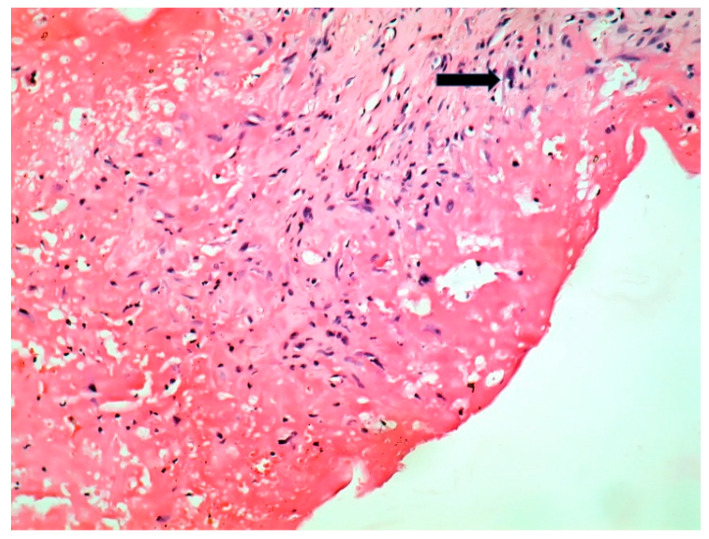
Tricuspid valve endocarditis; a multinucleated cell (arrow) within the mild inflammation at the vegetation base (arrow) (HE stain × 10).

**Figure 5:**
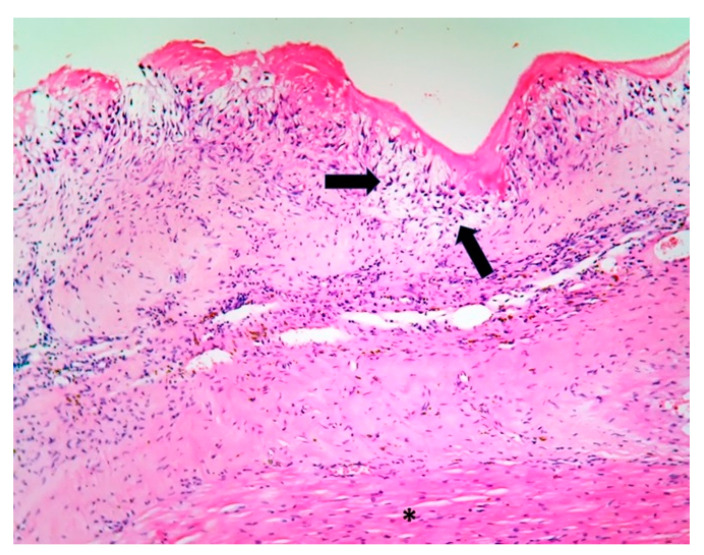
Endomyocardial fragment with mural endocarditis (the asterisk marks the myocardium), giant and foamy cells (arrows). * Special stains did not identify microorganisms (HE stain × 10).

**Figure 6:**
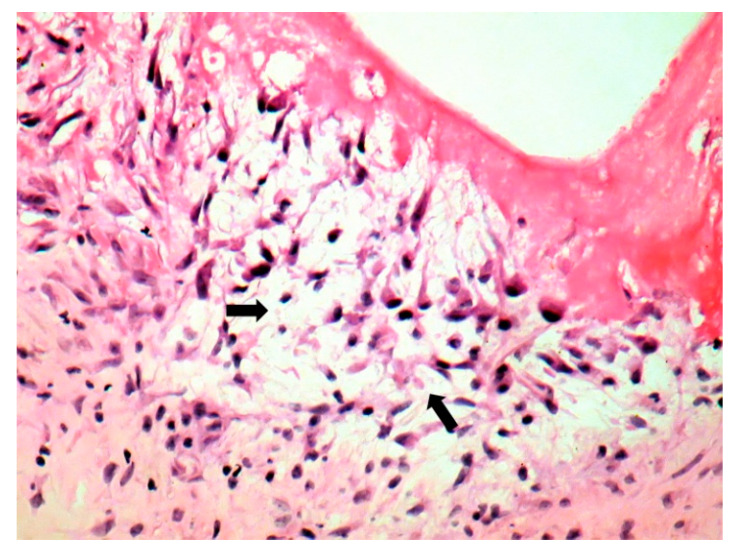
Foamy cells (arrows) (HE stain × 40).

**Figure 7:**
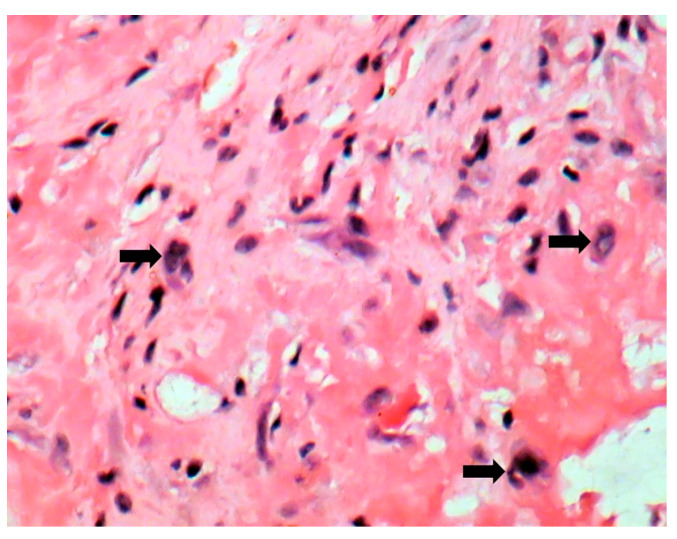
Giant cells (arrows) (HE stain × 40).

**Figure 8:**
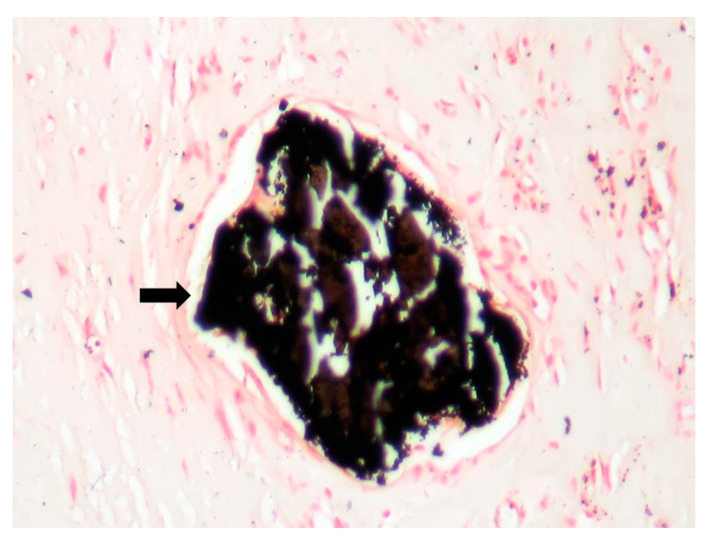
Calcification, black in Von Kossa special stain (Von Kossa stain × 20).

**Figure 9:**
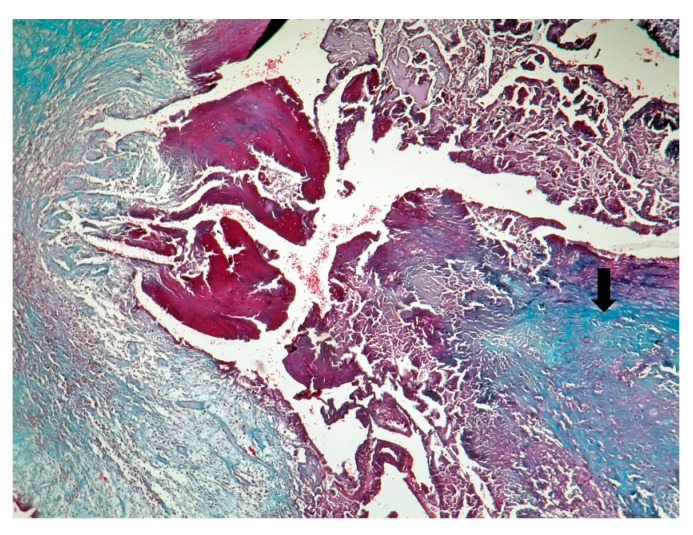
Fibrosis involving the valve and a part of the vegetation- arrow (Tricrom Masson stain × 5).

The patient underwent tricuspid valve replacement with biological prosthesis, on open heart surgery. The procedure was performed on beating heart, under circulatory assistance. The right atrium and ventricle were found to be severely enlarged. The tricuspid valve was completely destructured and giant vegetations replaced the cusps. The valve histology showed a tricuspid valve with multiple reparative changes. Giant, multinucleate and foamy cells and siderophages after repeated hemorrhages were noted (Figure 3, Figure 4, Figure 5, Figure 6 and Figure 7). Also, neoformation vessels with subocclusive intimal hyperplasia were found as well. A mural endocarditis was also present (Figure 5). Calcification and fibrosis involving the valve and the vegetation were observed (Figure 8 and Figure 9).

She received postoperatively short-term dobutamine, antibiotic prophylaxis (imipenem/cilastatin and vancomycin), anticoagulation with heparin, then acenocoumarol associated with low-dose aspirin, and dexamethasone was replaced by prednisone, resulting in complete correction of thrombocytopenia and general improvement. After all systemic signs of infection regressed, the patient experienced a BD articular flare, controlled by azathioprine dose escalation, along with doxycycline and hydroxychloroquine to be taken for at least 18 months, aspirin and statins.

## 3. Discussion

BD is a systemic vasculitis evolving with recurrent oral and genital aphthae, ocular disease, skin lesions, gastrointestinal involvement, neurologic disease, vascular disease and arthritis [5]. BD may involve vessels of any type and size and lacks specific antibodies [5]. BD cardio-vascular involvement is a combination of vasculitis, thrombosis and aneurysms [8]. BD may result in pericarditis, endocarditis, intracardiac thrombosis, myocardial infarction, endomyocardial fibrosis, or cardiac aneurysms [9]. Non-bacterial thrombotic endocarditis is a rare and seldom inaugural manifestation of BD [10]. This type of endocarditis in BD involves mainly the right heart valves, and right heart thrombi should generally raise the suspicion of BD [8,9]. The differential diagnosis in this setting includes myxoma, fibroelastoma or malignant tumors [11,12]. Other causes of tricuspid or pulmonary endocarditis, such as intravenous drug use, interatrial or interventricular communications, intracardiac devices or central venous catheters, were not present in our patient [4].

As would have been expected, no peripheral signs of subacute bacterial endocarditis, such as petechiae, Osler’s nodes, Janeway lesions, subungual or splinter hemorrhages, Roth spots, clubbing or fingers and toes, were present. These are generally seen in left-sided endocarditis. Right -sided endocarditis may manifest with respiratory signs- cough, hemoptysis, dyspnea, chest pain- reflecting pulmonary abscesses or embolism, with paradoxical emboli in the presence of intracardiac communications [4].

The prevalence of APL (mainly ACL and Aβ2GP1) is increased in BD [7]. Nevertheless, the propensity to thrombosis in BD is due to inflammation and endothelial cell dysfunction rather than to thrombophilia [7]. Azathioprine reduces thrombosis prevalence more than anticoagulation, which may be harmful with co-existing pulmonary aneurysms [4,7]. 

*C. burnetii* is an increasingly identified pathogen, mostly in culture-negative endocarditis. Most cases of acute *C. burnetii* infection resolve spontaneously, while others progress to chronic disease. As in our case, the co-existence of Q fever endocarditis with other superimposed infection has been described [13]. Also, *C. burnetii* has been reported to infect a left-sided intracardiac thrombus, mimicking an atrial myxoma [14]. *C. burnetii* may be discovered after long-time silent evolution, and various *Coxiella* antigens components may be demonstrated in the human host for years [15].

A phase I anti-*C. burnetii* IgG titer of 1:800 is generally accepted for the diagnosis of chronic Q fever and is included in the modified Duke criteria for endocarditis diagnosis [16]. Nevertheless, smaller anti-phase I titers were associated with chronic Q fever in Europe [17]. The paradigm in distinguishing between acute and chronic Q fever has recently shifted from relying on elevated phase I antibody titers [18,19,20]. As the serologic response is strain-dependent, the diagnosis of persistent infection is based upon persistence of clinical symptoms over 3 months, demonstration of an organic lesion (focus), and laboratory evidence of *C. burnetii* infection [18].

*C. burnetii* infections evolve with APL antibodies, which could aid to the diagnosis of the *Coxiella* endocarditis [2]. In acute Q fever ACL IgG antibodies were independently associated with endocarditis identified by TTE in patients with no previous valve involvement [18]. In the presence of ACL titers over 60 UGPL, mostly in men over 40 years, TEE (*transesophageal echocardiography)* should be performed to detect a clinically silent endocarditis in this setting [18]. *A. defectiva* infections are rare, inflicting mostly immunodepressed patients and previously affected valves [19]. The clinical course is slow and indolent, with a high rate of recurrence and cardiac and systemic complications such as hemophagocytic syndrome [19,21,22]. *A. defectiva* infections such as cerebral abscess, meningitis, osteomyelitis and septic arthritis have been described as well [21].

The type of valve involvement and histology in *C. burnetii, A. defectiva*, APL and BD are different (Table 1), but they can overlap and enhance each other. Histiocyte aggregates or clusters of multinucleated giant cells in the valves should always rise the suspicion of Q fever [23].

In chronic Q fever endocarditis the vegetations are small and sometimes absent, and the lack of visualization by TTE or TEE does not rule out a *C. burnetii* infection [13]. By contrast, *A. defectiva* usually forms large vegetations, as in our case [21]. The partial filling defect in the pulmonary left and right lobar arteries may have been due to BD, secondary APL or to infection, by either *A. defectiva* or *C. burnetii*.

ANA are not a classic finding in BD and may be found in BD with variable, but generally low prevalences, not related to disease activity [6,35,36]. Positive ANA can be found in intracellular infections as well [37]. Of interest, autoantibodies in BD may specifically target a recently identified autoantigen, CTDP1 (RNA polymerase II subunit A C-terminal domain phosphatase) ubiquitously expressed in the nucleus and cytoskeleton [5]. Moreover, CTDP1 contains a highly conserved haloacid dehalogenase (HAD) domain, similarly with that of the *C. burnetii* effector peptides CBU1676 and CBU0885 [38]. Thus, it it tempting to speculate that the ANA occurrence in our patient may have been due to cross-reactivity with *C. burnetii*.

Patients with *C. burnetii* endocarditis may not develop an efficient cellular response against the microorganism [23]. Multinucleated giant cells in Q fever endocarditis, as in our patient (Figure 4 and Figure 7), suggest an inefficient response to the bacterium [23]. Interleukin-10 (IL-10) impairs the inactivation of *C. burnetii* in macrophages [39]. Pre-existing valvular and vascular diseases, immunocompromised status, as well as genetic determinants in innate immunity, favor *C. burnetii* persistence [40].

Interesting, our patient had a flare after *C. burnetii* treatment. In BD activation of neutrophils and NLRP3 inflammasome (nucleotide-binding domain, leucine-rich containing family, pyrin domain-containing 3) is seen [41,42]. Nevertheless, *C. Burnetii* inhibits the NLRP3 inflammasome and also IL-17, important in neutrophil recruitment in BD [40,43,44,45]. Of interest, a similar tolerance induction using a recombinant Cholera toxin B subunit (conjugated with a peptide derived from the HSP protein 60) was therapeutically tried in BD uveitis [46]. 

Thrombocytopenia is a non-specific finding in fastidious germs endocarditis, as it was described in *C. burnetii* (in 39% to 65% of cases) [13,47], in *A. defectiva* endocarditis [47] and in BD as well [7]. Infective thrombocytopenia may occur through multiple mechanisms, and is related to decreased platelet formation or impaired survival [48]. Disturbance of thrombopoiesis through cytokines or thrombopoietin, antibodies against megakariocytes, platelet activation resulting in microvascular thrombi, organ sequestration or their phagocytosis, as well as cytotoxicity and apoptosis induced by bacterial products may all be involved [48,49]. Confounding causes include heparin-induced thrombocytopenia or transient postoperative thrombocytopenia related to bioprosthetic valves [50]. In our case the thrombocytopenia, although improved after anticoagulation, resolved only after surgery, likely related to the removal of pathogen source [50].

Identification of cerebral involvement during an infection is important prior to valve replacement, since both *C. burnetii* and *A. defectiva* have been described as causes of brain abscesses [21,51,52]. As in other types of cardiovascular surgery, the selective screening and elective repair, as opposed to emergency surgery, improve the prognosis [53].

Possibly the sources of *C. burnetii* in our patient were unpasteurized dairy products. For the *A. defectiva* infection, an oral source is likely, as *Streptococcus* and *Staphylococcus* infections are more prevalent in the oral flora of BD [42]. Apart from lifestyle change (including avoidance of passive smoking and control of cardiovascular risk factors such as arterial hypertension, dyslipidemia, weight and glycemic control [54], cardiovascular prophylaxis in patients with BD should take into account avoidance of unpasteurized dairy products and also of farm visits.

## 4. Conclusions

Intracellular infections may modify the presentation of autoimmune diseases. Endocarditis with “stealth pathogens” such as *C. burnetii* in BD is rare and possibly underdiagnosed. In a BD patient, ANA and APL positivity could warrant a search for *Coxiella* infection.

## Figures and Tables

**Table 1:** Comparative valve pathology in BD, APL, *A. defectiva* and *C. burnetii*.

	Behçet’s Disease	Antiphospho Lipid Syndrome	*A. defectiva*	*C. burnetii*
Macroscopic	Mural trombi, Endocardial fibrosis [23] Monovalvular involvement Mixoid degeneration Valve ulceration or fibrinous mass No fibrin valve deposits [24,25]	Small, warty, sessile vegetations on valve flow surface [26]	Large or small vegetations on valve flow surface [27]	Small or absent vegetations, with a smooth nodular aspect [13]
Microscopic	Mixoid degeneration, mixed inflammatory pattern, granulomas, thickening of the small vessels wall [23]	Platelets, fibrin, red blood cells Repetitive process with fibroblastic organization and neovascularization [28]	Fibrin, polymorphonuclear inflammation and some bacterial colonies [29]	Minimal mononuclear inflammation, histiocytes, foamy macrophages and mild vascularization [30] Granulomas [31] Microabscesses; usually no microorganism, sometimes coccoid inclusions [23] Fibrosis, thrombosis and calcifications, mimicking degenerative damage [32]
Predilection	Right heart [13]	Mitral, aortic, tricuspid valves [27]	Aortic, mitral valve [33]	Aortic, mitral valves [32]
Mechanism	Vasculitis, endothelial cells disruption, thrombo-inflammation, fibrinogen oxidation, hypercoagulability, APS induction, hyperhomocysteinemia, deficient fibrinolysis [34]	Valve endothelium initially normal [25] Fibrin-platelet, red blood cells thrombi [28]	Fibronectin adherence [35]	Vasculitis, APS induction [36]

## Data Availability

The data presented in this study are available on request from the corresponding author. The data are not publicly available due to privacy restrictions.
